# The value of an ecological approach to address micronutrient malnutrition

**DOI:** 10.1016/j.ajcnut.2025.07.006

**Published:** 2025-07-11

**Authors:** Daniel J Raiten, Jose M Saavedra, Gerald F Combs, Omar Dary, Emily Levin, Andrew A Bremer

**Affiliations:** 1 Office of Nutrition Research, National Institutes of Health, Bethesda, MD, United States; 2 Johns Hopkins University School of Medicine, Baltimore, MD, United States; 3 College of Human Ecology, Cornell University (Emeritus), Ithaca, NY, United States; 4 Private advisor in public health nutrition, Reston, VA, United States

**Keywords:** malnutrition, micronutrients, nutrition, nutritional ecology, nutrient deficiency

## Abstract

The global public health context has become increasingly complex with the confluence of widespread infectious and noncommunicable diseases, and widespread food insecurity, which has led to alarming rates of hunger and multiple consequences of malnutrition. A key component of this complexity is prevalent deficiencies of micronutrients (MNs, i.e., essential vitamins and minerals), many of which have been overlooked in public health programs addressing malnutrition. Here, we lay out an approach to more comprehensively address this complexity by considering the interactions of internal (biology, health, and developmental context) and external (diet/food environment, social, economic, climate, and physical) environments on humans and their nutritional health, i.e., an ecology. We present a conceptual framework for applying this ecological perspective with foci on the following: *1*) how to apply this approach to assessments that capture the complexity of the nutritional ecology; and *2*) how to expand the understanding of the biology of MNs in biological systems and the environmental factors that influence them. Suggestions are included for a targeted clinical, public health, and research agenda to inform the development of context-specific nutritional assessment and interventions.

## Introduction

Macronutrients (protein, fats, and carbohydrates) provide energy and support the development and maintenance of body structures and functions. Because of their relatively large quantitative needs, they are often the main foci of food-based approaches to addressing hunger (see Text Box 1) [1].

Text Box 1 **FAO Definition of Hunger**

Hunger is an uncomfortable or painful physical sensation caused by insufficient consumption of dietary energy sources. It becomes chronic when the person does not consume a sufficient amount of calories (dietary energy) on a regular basis to lead a normal, active, and healthy life. For decades, FAO has used the “Prevalence of Undernourishment” indicator to estimate the extent of hunger in the world; thus, “hunger” may also be referred to as undernourishment [1].

Alt-text: Text Box 1

Meeting macronutrient needs (i.e., establishing energy/food sufficiency); however*,* does not ensure nutritional adequacy. This is evidenced by the growing recognition of the paradoxical double burden: of obesity/diet-related noncommunicable diseases (NCDs) and micronutrient (MN) imbalance, shortages of essential MNs, which can occur under a variety of circumstances and particularly under conditions of food/nutrition insecurity [2,3].

As covered in a textbook devoted to the topic of vitamins’ biology [4] attempts to classify MN according to their functional impacts [5], and the reports developed under the aegis of the Biomarkers of Nutrition for Development (BOND) project [6], assessment of MN status presents a number of capacity and technological challenges. Moreover, because MNs function in multicomponent metabolic systems, the impacts of their deficiencies or excesses may not be readily apparent. Therefore, MN attention has been underrepresented in public health agendas, which is reflected in the term “hidden hunger,” now part of the global health lexicon [7].

Although both MNs and macronutrients present challenges to the public health enterprise, MNs are uniquely defiant due to limitations in our understanding of the factors (dietary, metabolic, physiologic, and environmental) that can affect their availability, absorption, utilization, function, physiological impacts, metabolic interactions, and prevalence of insufficiencies/excesses in specific populations. Addressing these issues necessitates being able to assess MN status and detect problems associated with MN deficiencies/excesses including their various etiologies.

To achieve these goals, we propose an approach – one that recognizes that people are subject to the same forces, interspecies, balances, and interactions that affect global ecosystems [8]. This means viewing humans as complex biological systems that interact with both internal (e.g., developmental stage, health status including therapeutic interventions, genetics, and microbiome health) and external (diets, food/water safety, sanitation, xenobiotic exposure, social/behavioral/economic, climate, and physical) environments. This view emphasizes the importance of context. Accordingly, this paper lays out a pedagogy for considering nutrition and health within an ecological framework. It focuses on nutrients essential for human health, particularly those that have been underrepresented in public health agendas. It differs from the traditional focus in public health nutrition, which has emphasized particular MNs for which clinical impacts have been obvious (e.g., vitamin A and iodine). Here, we present a different focus: one that employs an ecological perspective to determine MN needs in a comprehensive, context-specific, evidence-based approach.

### The global context implications for MN assessment

Text Box 2 includes data from the WHO/FAO that represent the current indicators of global nutrition, but without mentioning MN inadequacies. As highlighted by Stevens et al. [9], any human may be at risk of having one or more MN deficiencies depending on the context, and hence be dissimilar in high-income countries compared with low-middle income countries (LMICs), and in subregions and subpopulations of each.

Text Box 2 **WHO/FAO State of Global Nutrition**

“In 2022, 738.9 million people faced hunger, 2.4 billion in 2022 were moderately or severely food insecure, and >3.1 billion lacked access to healthy diets. The COVID-19 pandemic added 120 million to the chronically undernourished. By 2030, an estimated 590.3 million will suffer hunger. Progress toward global nutrition targets is uneven [10].”

Alt-text: Text Box 2

Although sufficiently alarming to warrant immediate and concerted action, these data do not recognize that whenever food is limiting, MNs may also be limiting. And thus, they make a compelling case for the need to address more than the macronutrients and the short list of MNs that have been the traditional focus of the global health agenda. This begs the question: which MNs? How do we know?

Addressing the complexity of multiple MN needs in the context of the complex global health scenario demands attention to the following 2 key components of translational science: *1*) approaching assessment in ways that capture the complexity of the nutritional ecology; and *2*) expanding understanding of the roles of MNs in biological systems and the environmental factors that affect them.

An additional issue is the potential unintended consequences of high MN intakes due to widespread, and frequently simultaneous, use of MN-delivery interventions (supplementation, food fortification). Such potential adverse outcomes may be consequent to health or nutritional status, gender and age [[11], [12], [13], [14]], which may affect individuals differently if they are healthy and well-nourished or not or if they are experiencing impaired metabolic protections, such as those that are malnourished and/or reside in infectious environments, and receiving or not medical treatment [15,16]. Excessive intakes may also affect the assessment of nutrient status of individuals and populations, the efficacy/safety of therapeutic drugs, and the status and physiological functions of other MNs [6,17,18].

Of additional concern is the fact that diet-related health prevalence data seldom include information about other conditions that can affect health. These include changes in “food environments,” [19] the colliding epidemics of both NCD and infectious diseases, and the growing impacts of climate and environmental changes [[20], [21], [22]].

With these considerations, the challenge in addressing MN status specifically or malnutrition, more broadly, is framed by the following trends:

1. Despite the significant progress made in reducing the impacts of undernourishment on child health, anemia and stunting/wasting continue to be major challenges for which the roles of MNs remain poorly understood.
2. The global prevalence of overweight/obesity and NCD has increased in all age groups and countries, including LMICs, which for the latter focus on undernutrition. The roles of MNs under these 2 conditions remain unclear [23,24].
3. Infectious diseases (COVID-19, HIV, malaria, tuberculosis (TB), diarrheal, and other tropical diseases) remain daunting public health concerns that target food/nutrition insecure groups, particularly those in LMICs [25]. The roles of MNs in the prevention/treatment of these diseases need to be elucidated.
4. A collision is occurring among infectious diseases, NCD, food insecurity, and excess/insufficient intakes of energy, protein, and/or MNs within populations, and in many settings, the same individuals [3,26].

To inform effective, context-specific, and safe interventions and guidance, approaches must focus not only on identifying nutritional problems but also on determining their etiologies. That challenge calls for a new pedagogy of public health nutrition.

That pedagogy must include a focus on the nature and level of information needed to be able to address these needs, both in individuals and populations to develop and support safe and efficacious interventions and programs. It must regard the ability to assess the nutritional status of individuals and populations as the basis for linking diet, nutrition, and health with ecological adaptation to survive the assaults of aggressive pathogens. Text Box 3 highlights some of the key features of nutritional status and its assessment [27,28].

Text Box 3 **Nutritional status is:**

• The operational measure of diet adequacy and the integration of dietary components to support health.
• Achieved as a result of a series of behavioral, physiological, and metabolic processes involved in the taking in and utilizing dietary substances/nutrients that support growth, repair, and maintenance of the body and its functions [27].
• Conceptually viewed as involving multiple nutrients, their potential interactions within biological systems.
• An illustration of the complexity of nutritional ecology with nutritional status as both an input and an outcome affecting and affected by the processes of nutrition.
• Only accurately assessed and interpreted in the context of the life cycle, health status, and other aspects of the nutritional ecology [28].

Alt-text: Text Box 3

Although the literature of the biology and assessment of specific MNs is substantial, global attention has been focused on a relatively limited number of nutrients: vitamin A, iron, folate, iodine, and zinc [6]. Yet, there is evidence of deficiencies of other MNs in several settings. Thus, new and additional methodologies as well as a more comprehensive tactical approach to nutrient assessment are needed to diagnose problems related to MN status and provide the information necessary to make judgments about their etiologies and health impacts. Achieving this goal will call for addressing the following questions:

• Why would we need to study >1 MN at a time?
• What is the nature of the role of nutrition within systems of interest that would support a multinutrient approach?
• How can we assess status and potential for problems within the systems of interest?
• How can we more precisely determine the essential data needed to ascertain the etiology of problems identified?
The following case study is the first of 2 presented here to illustrate the need for integrated approaches to addressing problems related to multiple MNs.

Case study 1: the “ecology of anemia” (and the metabolic management of iron status). Applying an ecological approach to anemia, a high-priority global health challenge [26].

#### Background

**FIGURE 1:**
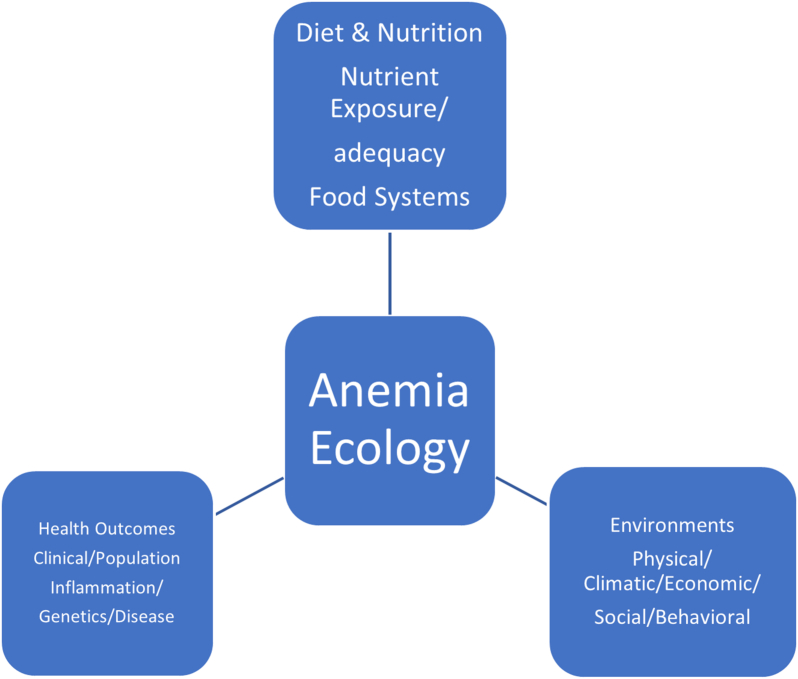
Components of the anemia ecology [31,32].

The prevalence of anemia and iron deficiency (ID) has been an intractable challenge for the global health community. Moderate and severe anemia contributes to increased morbidity and mortality, particularly in women of reproductive age and preschool-age children [29]. However, the real magnitude of these problems is uncertain because of errors in the measurement of blood hemoglobin concentration [30] and, hence, anemia prevalence estimates, as well as an inadequate appreciation of the multidimensional nature of anemia and iron status. Consequently, and despite concerted efforts, the WHO 2025/2030 target for reduction of anemia in women of childbearing age remains elusive. Text Box 4 highlights some prominent prevalence data along with an important caveat. Figure 1 presents the components of the anemia ecology [31,32].

Text Box 4 **Global estimates of anemia**

There were a total of 1.93 billion people with anemia in 2013 [33]:

• 950 million cases of mild anemia,
• 906 million cases of moderate anemia, and,
• 75.6 million cases of severe anemia
• However, as mild and moderate anemia have similar numbers of individuals, it may reflect a large random variation in the determination of the hemoglobin concentration because of the use of drops of capillary blood, mostly in LMIC [30].

Caveat: most of this information comes from Demographic and Health Surveys (DHS's) and similar population surveys. Those figures may be overestimated. For example, for children aged <24 mo, the WHO has recently recognized that the threshold for anemia diagnosis was higher (11 g/dL) than the current recommendation (10.5 g/dL) [34], making the actual prevalence of anemia in this higher than previously recognized.

Alt-text: Text Box 4

The biological mechanisms underlying anemia have been reviewed [35]. **Text** Box 5 shows the components of its etiology.

Text Box 5 **Etiology of anemia**

Anemia is the condition of insufficient circulating levels of red blood cells containing hemoglobin-bound iron both in sufficient amounts to meet the oxygenation needs of tissues.

Anemia can develop when:

○ Red blood cells contain insufficient iron to meet tissue oxygenation needs (insufficient dietary iron, infection, inflammation);
○ Red blood cell production is limited (insufficient iron supply; functional deficiencies of iron, folate, vitamin B_12_, or vitamin A);
○ Red blood cells are lost (e.g., menstruation and wounding) or destroyed (parasitism causing intestinal bleeding);
○ Iron stores are insufficient to support adequate hemoglobin production, whereas other MN are reduced or absent (folate, vitamin B_12_, vitamin A). Other MN deficiencies are less frequent causes of anemia overall, but they can be important in some settings.
○ The synthesis of hemoglobin and/or red blood cells may be in response to pathogens.

Alt-text: Text Box 5

Considerations regarding ID and anemia: Detection of anemia depends on measurement of a decline in the circulating hemoglobin concentration below thresholds based on levels needed to maintain adequate tissue oxygenation. Although there are multiple causes of anemia, ID remains a primary cause. Critical to our ability to address the role of iron is the recognition that ID may not only be due to low iron stores (i.e., “absolute ID”) because of inadequacy (insufficient iron intake) and/or low bioavailability, but it can also be due to impaired iron metabolism (“functional ID”). Significant advances have been made in our understanding of the mechanisms underlying ID as well as its interactions with other causes of anemia [36].

The common perception about the predominance of dietary iron inadequacy conflates absolute ID with functional or impaired iron physiology due to other causes, thus begging the following questions: how to distinguish between the contribution of absolute (nutritional) ID from those of other factors? How to account for the multiple factors that may contribute to anemia in a population? The ecological approach emphasizes the coincidence between regions with high prevalences of anemia attributed to ID (both absolute and functional), those with endemic infections, and those with widespread genetic conditions affecting red blood cells, especially in sub-Saharan Africa and regions in Asia and Oceania.

The utilization of iron is entwined with the metabolism of other MNs (thiamine, riboflavin, and pyridoxine have been linked with iron and anemia), and multiple MN deficiencies can contribute to anemia. Therefore, addressing anemia requires the application of new and more integrated approaches to ascertain the roles of individual/multiple nutrients in cases of anemia [[36], [37], [38], [39], [40], [41]].

#### Assessment

**FIGURE 2:**
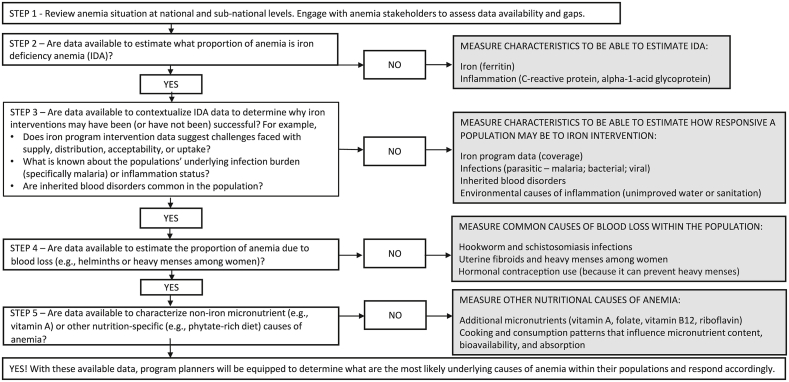
A framework for assessing anemia in clinical and population-based settings. Reproduced from Williams et al. [32] in accordance with the Creative Commons license CC BY 4.0.

To make progress on global goals to reduce anemia and to determine with better accuracy the effectiveness of any interventions, it is imperative to have appropriate assessment tools not only for blood hemoglobin concentrations but also for the underlying causes of anemia in both clinical and population-based settings. Figure 2 shows a presentation of a potential framework for assessment [32].

#### Summary

Both anemia and ID are multifactorial conditions that require assessments more complete than simply detection by low hemoglobin concentration. Although sensitive to changes in nutrient status, neither hemoglobin nor serum ferritin provides etiologic insight without additional information. The ability to determine the prospective roles of the other nutrients will call for understanding the patient’s internal and external environments. Both anemia and ID represent ideal candidates for the ecological approach, which will facilitate the development of a more complete understanding of the factors contributing to anemia and other priority public health problems [[42], [43], [44], [45]].

### Expanding understanding of MNs in biological systems

Nutritional assessment should address multiple nutrients and their interactions in biological systems [46]. This may be facilitated by the use of “omics” techniques, i.e., the simultaneous determination of multiple genes, proteins, or metabolites. These methods have changed research foci from specific metabolic pathways to complex physiological systems.

Systems approaches in nutrition research [[47], [48], [49]] have enhanced understanding of the interplay of genes and nutrients in metabolism and their relevance to health. Here, the term “nutriomics” is adapted to refer to the study of patterns (i.e., “nutriomes”) of nutrient functions and interrelations in biological systems of interest. An illustrative example of this approach has been employed in the study of neural networks in the human brain “connectme” [[50], [51], [52]]. Another example is the description of the nutriome of inflammation [53], which has revealed multiple nutrient-dependent points within this critical system. Such patterns raise research questions:

• How do particular nutrients interact to affect the function of the system?
• How sensitive is the system to fluctuations in particular nutrients?
• Is there a level of plasticity that allows for physiological/homeostatic control of nutrients within the system to ensure its function even in times of interrupted supply/exposure, e.g., hepcidin control of iron absorption during inflammation?
• To what extent is the function of MN with a given system affected by endogenous factor, e.g., toxins, therapeutic drugs, and environmental stressors?

Metabolomics, the measure of metabolites in biological specimens [54], offers potentially valuable approaches for improving understanding of MN functions [55,56]. Although valuable in characterizing the nature of diet and metabolic function under conditions of health and disease, absent ecological data, they lack the ability to provide insight into the etiologies of diet-related health problems [57,58].

The complexity and value of addressing the intersection between nutrition, the microbiome of the human gut, and its ecology were the subject of a recent review. The following case study provides a deeper appreciation of the nature and value of the ecological approach applied to these complex systems [59].

Case study 2: microbiome, MN, and enteric environmental dysfunction (EED). Applying microbiomics to the study of a priority public health issue.

#### Background

The last couple of decades have seen an explosion in understanding of the human microbiome, the complex microbial ecosystem associated with and interacting with the host, particularly in the gut. The environment microbiota, a broad collection of bacteria, viruses, and fungi, offer early-life exposures to factors that shape an individual’s microbiome [60]. The gut microbiome is fundamental in maintaining a healthy digestive function, “training” the host immune system, and in modulating host metabolic responses to a variety of internal and external exposures. The gut microbiota are affected by several factors, including mode of birth, level of microbial exposure, sanitation, use of antibiotics, and living environment. These factors can modify the microbiome in ways that affect health. Disruptions of a “healthy microbiome,” particularly in early life, have been associated with such chronic conditions as infection, allergies, autoimmune disease, and obesity [[60], [61], [62]].

#### Enteric environmental dysfunction

**FIGURE 3:**
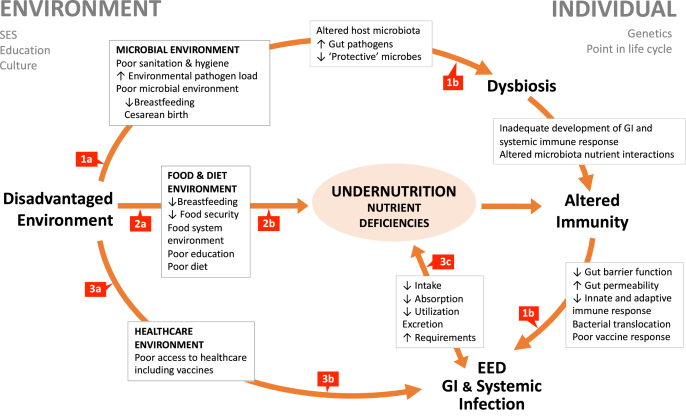
Relationships between a disadvantaged environment, gut microbiota, gastrointestinal function, and nutrition. Blue boxes indicate environmental etiologic mediators. Green boxes etiologic mechanisms in the individual. Red numbered boxes indicate potential interventions addressing etiologic mediators and mechanistic targets. 1a: optimize the development of healthy gut microbiota: avoid unnecessary cesarean births, breastfeeding, water and food sanitation and hygiene; 1b: prevent or repair dysbiosis beyond sanitation efforts with microbiome-directed interventions. For example, prebiotics, probiotics, antimicrobials; 2a: Modify food systems to improve food security, making foods available, accessible, and affordable, including breastfeeding, diet, and nutrition education; 2b: identify and provide specific nutrients via supplementation and fortification. For example, identifying specific target nutrients and populations; 3a: improve preventive healthcare access; 3b: improve therapeutic healthcare access for the management of GI and other infections; 3c: nutritional management of EED and infectious disease. This requires identifying increased requirements for acute and chronic inflammatory processes and managing poor intake and decreased nutrient absorption. EED, enteric environmental dysfunction; GI, gastrointestinal.

EED is an example in which an ecological perspective aids in understanding the etiology of malnutrition, its relationship with the environment, and the impact of potential interventions. EED is a subclinical state of an altered gut microbiome and chronic intestinal inflammation, with changes in gut permeability and immunity. It is understood to be a causal factor connecting poor sanitation and malnutrition, particularly stunting, and poor MN status [[62], [63], [64]]. Three key environmental conditions found in economically disadvantaged settings are conducive to EED: inadequate diet/food availability; inadequate/poor sanitation; and limited access to preventive and therapeutic health care. Figure 3 depicts the relationships between these environmental etiologic drivers, gut microbiota, gastrointestinal (GI) function, and nutrition.

An infant’s microbiome is established in early life and is shaped by the environmental microbial conditions (sanitation, hygiene, and birthing practices), and early diet (breastfeeding, safe water, and food). Poor microbial environmental and diet conditions lead to dysbiosis (altered gut microbiome) that can start at birth and persist through childhood. The consequences include compromised immunity and GI functions, often subclinical and sometimes punctuated by acute GI infection, decreasing nutrient absorption and utilization. Once established, dysbiosis and immunologic dysfunction will persist, increasing risk of recurrent infections, as well as vaccine failure, perpetuating undernutrition. Persistent subclinical dysbiosis leads to persistent inflammation, contributing to stunting [65,66] and gut dysfunction, affecting the risk of, and resilience to, malnutrition [64,65,67,68].

A critical dynamic: MN and the microbiome: The microbiome also affects the availability of MNs to the host. Gut microbes produce some vitamins (e.g., K and the B-vitamins) and use most vitamins and minerals in their own metabolisms. They can increase the bioavailability of some MN minerals (e.g., calcium, phosphorus, zinc) by removing diet-sourced anti-nutritional compounds (e.g. phytates and polyphenols) that would otherwise bind and render them nonabsorbable.

It is not clear how the net utilization of MNs by the gut microbiota may be affected by dysbiosis and EED. Inadequate protein and MN intakes (particularly vitamin A and zinc) directly affect gut epithelial integrity and immune responses, increasing susceptibility to GI infection, reducing nutrient utilization, and reducing MN status. Evidence also indicates that, once established, EED (characterized by increased intestinal permeability and inflammation) becomes a risk factor for MN deficiencies, particularly of vitamin A, pyridoxine, calcium, and zinc, independent of dietary intake and systemic inflammation. MN interventions may reduce EED and its consequences; however, control of MN deficiencies may require the direct specific management of dysbiosis and EED [69,70].

EED is also associated with an increased risk of absolute ID, requiring an appropriate supply of this MN fortification or supplementation [71]. However, iron is also essential for most pathogenic bacteria. Studies have found that iron supplementation can exacerbate dysbiosis, increase gut inflammation and permeability, and increase the risk of infection with pathogens able to utilize iron [72,73].

These effects need to be considered in assessing current MN interventions, because iron may contribute to decreased effectiveness. Opportunities to circumvent such drawbacks of dietary iron include the use of more readily absorbable iron sources and supplementation with pre- or probiotics to modulate the microbiome and decrease inflammation [64,72,73].

#### Assessment

Addressing malnutrition, particularly stunting and MN deficiencies, especially in LMIC settings, requires an understanding of the impacts of poor microbial environments on the microbiota. This will better inform assessments and intervention plans. It is likely that improvements in sanitation may yield the best outcomes when dysbiosis is addressed early, starting at birth, before the establishment of EED. New technologies to characterize human microbiota and assess their functions and host interactions are likely to lead to better ways to support nutrition and health [58,74].

In conclusion, the global health context is complex, as is its influence on the ability to assess the role of diet/nutrition in health. Although much has been learned about this complexity, a compelling case can be made for a new approach that is capable of identifying diet/nutrition-related health problems while also elucidating their etiologies. As illustrated in the 2 case studies, a need exists to employ this more comprehensive and inclusive approach to learn the roles of MNs in biological systems relevant to health. Improving the precision and efficacy of clinical care and health surveillance will require more complete understandings of the contexts of nutrient deficiencies, as can be facilitated through the use of ecological approaches as outlined here. The challenges are great, but the tools are available. Their use can advance understanding of the health roles of diet/nutrition and inform context-specific means of addressing persistent problems of public health.

## Author contributions

The authors’ responsibilities were as follows – DJR: the original idea for the perspective and wrote the initial draft; JMS, GFC, OD, EL, AAB: provided intellectual input and contributed to the writing of subsequent manuscript drafts; and all authors: reviewed and approved the final manuscript.

## Disclaimer

The contents of this article represent the authors’ views and do not constitute an official position of the NIH, USAID, or the United States Government.

## Data availability

Not applicable.

## Funding

The authors report no funding received for this activity.

## Conflict of interest

The authors declare no conflict of interest.
